# Correlation between component alignment and short-term clinical outcomes after total knee arthroplasty

**DOI:** 10.3389/fsurg.2022.991476

**Published:** 2022-10-13

**Authors:** Yichao Luan, Min Zhang, Tianfei Ran, Huizhi Wang, Chaohua Fang, Maodan Nie, Min Wang, Cheng-Kung Cheng

**Affiliations:** ^1^ School of Biological Science and Medical Engineering, Beihang University, Beijing, China; ^2^ Department of Orthopaedics, Xinqiao Hospital, Army Military Medical University, Chongqing, China; ^3^ School of Biomedical Engineering, Shanghai Jiao Tong University, Shanghai, China; ^4^ Department of Joint Surgery, Ningbo No.6 Hospital, Ningbo, China

**Keywords:** total knee arthroplasty, component alignment, clinical outcomes, outlier rate, linear correlation

## Abstract

**Objective:**

This study aimed to investigate the correlation between component alignment and short-term clinical outcomes after total knee arthroplasty (TKA).

**Methods:**

50 TKA patients from a regional hospital were enrolled in the study. The following component alignments were measured from radiological data acquired within 1 week after surgery: hip-knee-ankle angle (HKA), medial distal femoral angle (MDFA), medial proximal tibial angle (MPTA), femoral flexion-extension angle (FEA), tibial slope angle (TSA), femoral rotational angle (FRA) and tibial rotational angle (TRA). The Hospital for Special Surgery (HSS) knee scoring system was used to assess clinical outcomes after 1 year, with patients being divided into three groups (excellent, good and not good) according to the HSS scores. Difference analysis and linear correlation analysis were used for the statistical analysis.

**Results:**

The results showed significant differences in MDFA (*p* = 0.050) and FEA (*p* = 0.001) among the three patient groups. It was also found that the total HSS had only a moderate correlation with FEA (*r* = 0.572, *p* < 0.001), but FEA had a positive linear correlation with pain scores (*r* = 0.347, *p* = 0.013), function scores (*r* = 0.535, *p* = 0.000), ROM scores (*r* = 0.368, *p* = 0.009), muscle scores (*r* = 0.354, *p* = 0.012) and stability scores (*r* = 0.312, *p* = 0.028). A larger MDFA was associated with lower FE deformity scores (*r* = −0.289, *p* = 0.042) and the TSA had a positive influence on the ROM (*r* = 0.436, *p* = 0.002). Also, changes in FRA produced a consequent change in the FE deformity score (*r* = 0.312, *p* = 0.027), and the muscle strength scores increased as TRA increased (*r* = 0.402, *p* = 0.004).

**Conclusion:**

The results show that the FEA plays a significant role in clinical outcomes after TKA. Surgical techniques and tools may need to be improved to accurately adjust the FEA to improve joint functionality and patient satisfaction.

## Introduction

Total knee arthroplasty (TKA) is the most effective treatment for severe arthritis of the knee joint. However, about 20% of patients are reportedly dissatisfied with the outcome because of joint pain or restricted function (1, 2). Malalignment of the knee prosthesis, which possibly results from inadequate determination of anatomical landmarks, the thickness of the saw blade and surgeon experience (3–5) have been reported as some of the main reasons for dissatisfaction and even revision (6, 7). Previous studies found that malalignment of knee prostheses can cause patellofemoral mal-tracking and incongruence with the femoral-insert interface, which may cause postoperative complications such as anterior knee pain and patellar subluxation (8, 9). The alignment of the prosthesis also influences the biomechanics and kinematics of the knee joint, such as stress on the ligaments, anterior-posterior translation of the femoral component and polyethylene wear (10–14).

Mechanical alignment is considered to create a biomechanically friendly environment in the knee joint that aims to position both the femoral and tibial components perpendicular to the mechanical axis. This method of aligning the components has been proven to produce good clinical and functional outcomes as well as long survivorship (15, 16). Some surgeons have suggested that the “safe zone” for the hip-knee-ankle (HKA) angle is with tibiofemoral alignment on the coronal plane being 180° ± 3° (17, 18). Patients aligned in this zone have reported better clinical outcomes (19), but, in contrast, some studies found no difference in outcomes or survivorship regardless if the alignment is within the “safe zone” (20, 21). Moreover, it has been reported that the “safe zone” may not be applicable to modern personalized alignment strategies (22). Complicating the discussion, differences in component alignment on the sagittal and transverse planes can also have a considerable impact on the joint. Some studies have shown that maintaining component alignment within 3° on the sagittal or transverse planes will not significantly affect the clinical outcomes (23, 24). However, opposing results have also been reported in other literature (25, 26). Few studies have examined the correlation between clinical outcomes and the alignment of knee components on all three planes (transverse, frontal and sagittal) in the same cohort of patients.

Recent advances in navigation, patient-specific instrumentation and robotics have improved surgical precision, but many surgeons still prefer conventional techniques because of the longer surgical time, higher cost and lack of qualifiable improvements in clinical outcome with the more advanced methods (27–30), a possible reason might be the greater attention to coronal alignment rather than also considering the sagittal and transverse planes, as well as unclear correlations between planar alignment and clinical outcomes. Therefore, this study aimed to investigate the correlation between the clinical scores and component alignment on all three planes (transverse, frontal and sagittal) using radiological measurements and clinical follow-up. It was hypothesized that there was a linear correlation between the alignment and clinical scores.

## Materials and methods

### Patients

This retrospective research was approved by our institutional ethics committee and all patients provided informed consent before involvement. This study retrospectively assessed all primary TKA procedures performed in a regional hospital between June 2019 and December 2020. In total, 276 TKA procedures were considered. The exclusion criteria of this study were as follows: (1) patients preoperatively diagnosed with rheumatoid arthritis or traumatic arthritis; (2) patients with served extra-articular deformities, trauma or other joint diseases; (3) patients treated with bilateral TKA; (4) patients imaged more than 1 week after the procedure; (5) patients followed up less than 1 year after surgery. After exclusions, 50 patients (Female:Male = 42:8) were enrolled in this study including 24 left knees and 26 right knees. The age of these patients was 68.40 ± 8.73 years old.

### Surgical procedures

All subjects were implanted with a posterior-stabilized (PS) knee system (Vanguard, Zimmer Biomet, USA) by an experienced senior knee surgeon following the approved guidelines for this prosthesis. The anteromedial incision and medial parapatellar approach were adopted for all TKA procedures and the prostheses were positioned using mechanical alignment. After cutting the distal femur, an intramedullary rod was placed into the femur along the anatomical axis. A valgus angle between the anatomical axis and mechanical axis was set according to pre-operative radiographs to ensure the cutting plane lay perpendicular to the mechanical axis. On the sagittal plane, the cutting line was perpendicular to the rod. The size of the femoral component was determined using the AP sizer and a 4-in-1 cutting block was placed when the slot was parallel to the trans-epicondylar axis (TEA). The location of the block was checked according to the anterior reference, and the distal femur was resected before the PS box was prepared following the surgical guidelines.

The extramedullary method was used for tibial resection. The cutting plane was perpendicular to the mechanical axis of the tibia on the coronal plane with a 5-degree slope on the sagittal plane. The size of the tibial component was confirmed following the best tibial coverage in both AP and medial-lateral (ML) directions. Rotational alignment of the tibial component was determined with the tibial trail under the knee flexion. The patella was repaired to an appropriate shape instead of the resurfacing.

### Radiographic assessment

**Figure 1:**
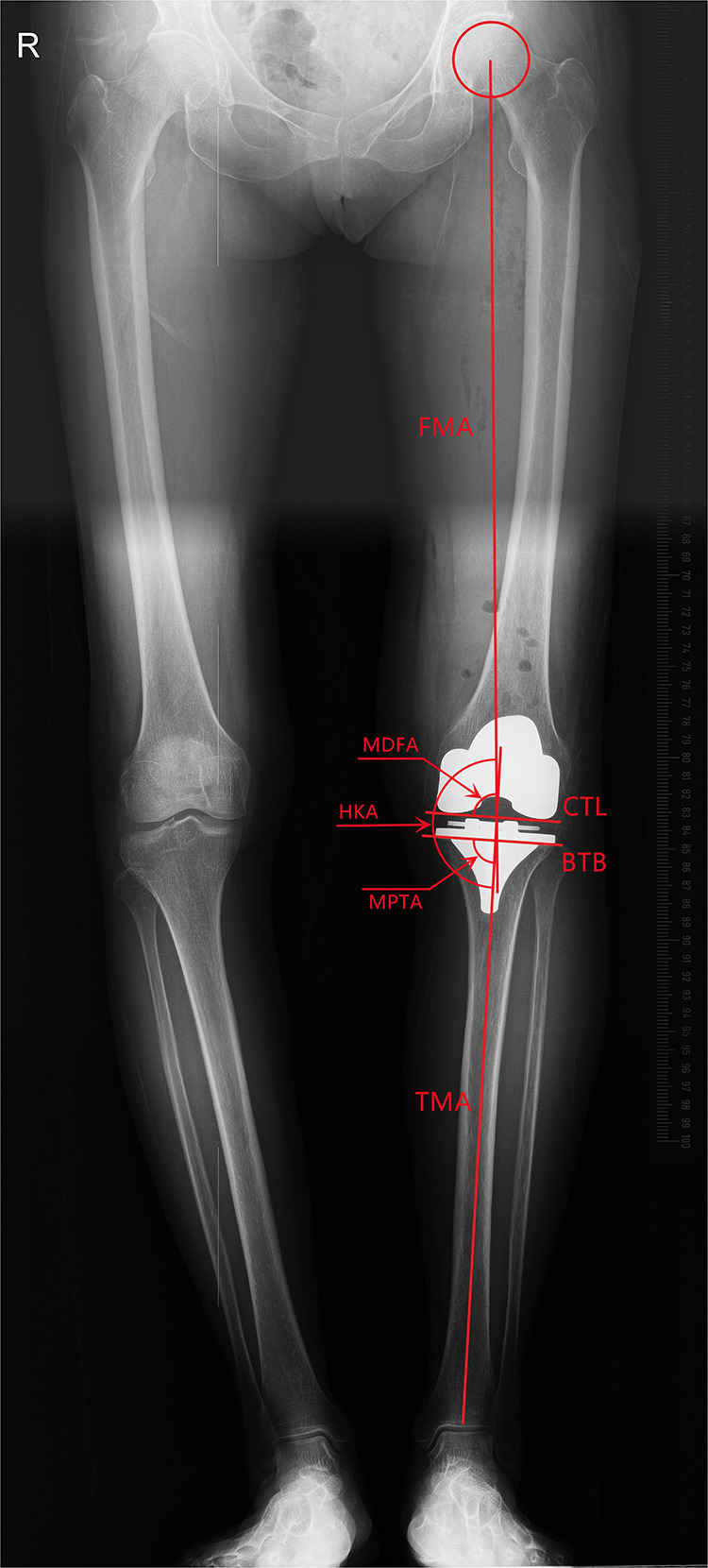
Measurement on the coronal plane (FMA, femoral mechanical axis: a line connecting the centers of the femoral head and knee joint; CTL, condylar tangent line; BTB, border of tibial baseplate; TMA, tibial mechanical axis: a line connecting the centers of the knee joint and ankle joint; MDFA, medical distal femoral angle; MPTA, medical proximal tibial angle; HKA, medical angle between FMA and TMA).

**Figure 2:**
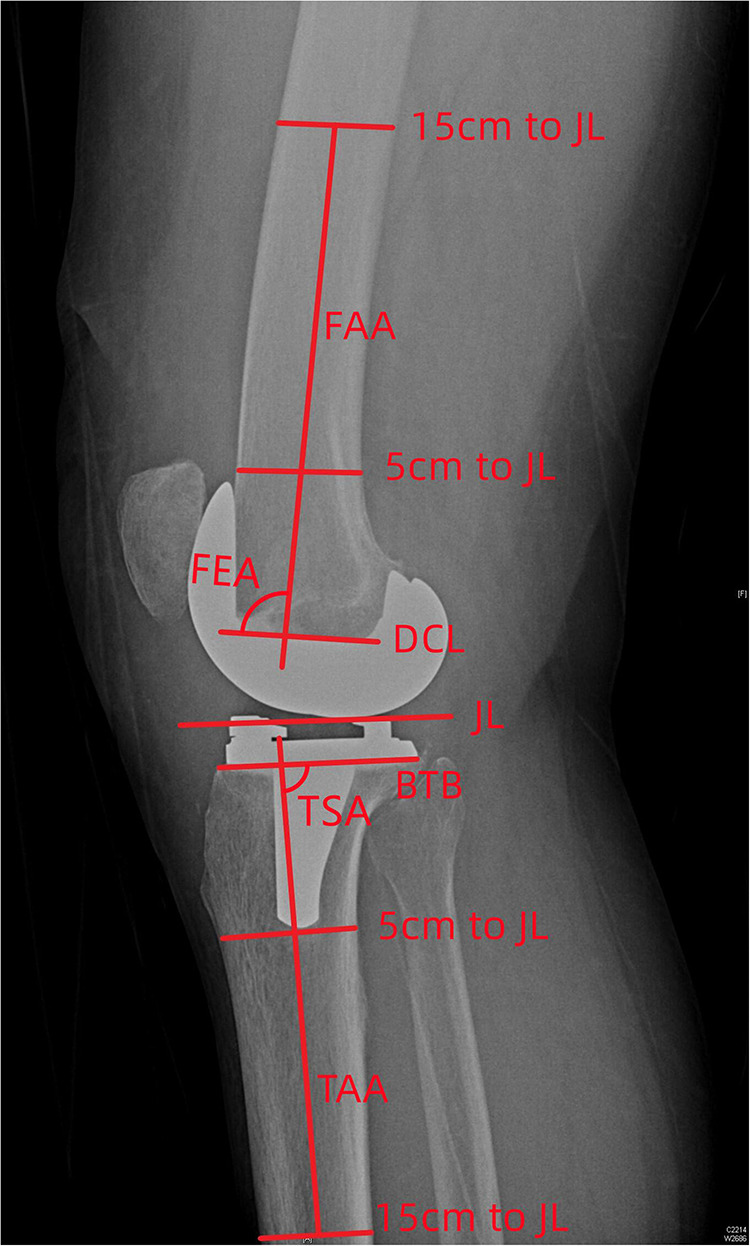
Measurement on the sagittal plane (JL, joint line; FAA, femoral anatomical axis; DCL, distal cutting line; BTB, border of tibial baseplate; TAA, tibial anatomical axis; FEA, femoral flexion-extension angle; TSA, tibial posterior slope angle).

**Figure 3:**
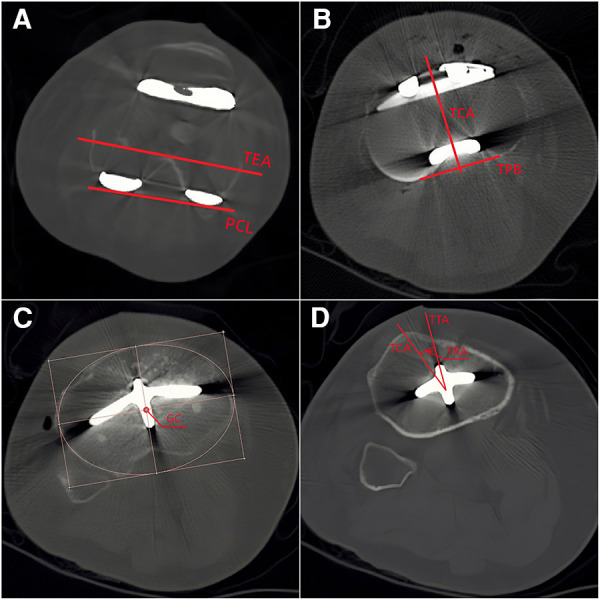
Measurement on the transverse plane (**A**: TEA, trans-epicondyle axis; PCL, posterior condylar line; **B**: TPB, tibial posterior border; TCA, tibial component axis: the mid-perpendicular line of the TPB; **C**: GC, geometrical center; **D**: TRA, tibial rotational angle).

Full leg weight-bearing anterior-posterior (AP) radiographs were taken preoperatively to measure the angle between the mechanical and anatomical axis, and lateral radiographs were used to estimate the required size of femoral component. The standard anteroposterior and lateral radiographs and the artifact-reducing CT images of the knee joint were taken routinely within 1 week after surgery. On the coronal plane, the hip-knee-ankle angle (HKA), medial distal femoral angle and medial proximal tibial angle were used to assess joint alignment (31). The femoral mechanical axis was defined as a line connecting the centers of the femoral head and knee joint, and the tibial mechanical axis was a line connecting the centers of the knee joint and ankle joint. The medial angle between the mechanical axis of the femur and tibia was taken as the HKA, the medial angle between the mechanical axis and condylar tangent line of the femoral component was MDFA, and the angle between the mechanical axis and the border of the tibial baseplate was the MPTA (Figure 1). The femoral flexion-extension angle (FEA) and tibial slope angle (TSA) were used to evaluate alignment on the sagittal plane (24), with the anatomical axis being the line that connected the midpoints of the outer cortical diameter at 5 and 15 cm proximal to the joint line on the femur and tibia (32). The anterior angle between the femoral anatomical axis and the distal cutting line was the FEA, and the posterior angle between the tibial anatomical axis and the border of the tibial baseplate was the TSA (Figure 2). On the transverse plane, the femoral rotational angle (FRA) and tibial rotation angle (TRA) were used to identify the alignments. The angle between TEA and the posterior condylar line (PCL) was FRA (Figure 3A). The TRA was determined by the tibial component axis (TCA) and tibial tuberosity axis (TTA) (Figure 3D). TCA was the mid-perpendicular line of the tibial posterior border (TPB) (Figure 3B). TTA was the line connecting the geometrical center (GC) (Figure 3C) of an ellipse of best fit around the proximal tibia just below the metal base plate and the center of the most prominent part of the tibial tuberosity (9). Neutral tibial rotational alignment was considered to be 18° internal rotation from TTA to TCA (33). Alignment errors were calculated as the difference between the preoperative surgical plan (HKA = 180°, MDFA = MPTA = FEA = 90°, TSA = 85°, FRA = 0°, TRA = 18°) and actual measurements from radiographs, and the outlier was considered as the errors more than 3° (17, 18). The outlier rate was the ratio of number of outlier cases to the total cases. The angles on the coronal (HKA, MDFA, MPTA) and sagittal (FEA, TSA) planes were measured by Picpick 5.0 (NGWIN, Korea) while the angles on the transverse plane (FRA, TRA) were measured by Mimics 21.0 (Materialise, Belgium). All measurements were recorded to an accuracy of 0.1°. Each angle was measured three times, with the average being considered the result for that angle.

### Post-operative follow-up and evaluation

All patients were followed up by physical examination in the outpatient department for at least 12 months after surgery. The Hospital for Special Surgery knee scoring system (HSS) was selected to assess the clinical outcome (34). The HSS score consisted of six sections, including pain (30 scores), function (22 scores), range of motion (18 scores), muscle force (10 scores), flexion-extension deformity (10 scores) and stability (10 scores). The maximum score achievable is 100 which is the sum of the six sections. The patients were divided into three groups according to their HSS scores. Scores between 85 and 100 were considered excellent (Group A), scores between 84 and 70 points were considered good (Group B), and scores less than 69 points were considered not good (Group C) (34).

### Statistical analysis

All statistical analyses were performed using IBM SPSS Statistics 26 (SPSS Inc, Chicago, USA). Power analysis was used to calculate the sample size. The power level was set as 80% with a 0.05 significance level, and the effect size was set as 0.44 (35). The analysis indicated that 43 participants were needed to provide a statistical power of 80%. The Kolmogorov-Smirnov test was used to test the normality of all data and data was regarded as a normal distribution when the significance was greater than 0.05. Quantitative data was expressed as a mean ± standard deviation. The linear correlation between joint alignments (HKA, MDFA, MPTA, FEA, TSA, FRA, TRA) and HSS scores, both the total score and section scores, was analyzed by Pearson analysis when the variable was distributed normally, and by Spearman analysis when the variable was not normally distributed. The correlation coefficient (*r*) assumes any value from −1 to 1, with an |*r*| value of less than 0.4 being considered a weak correlation, moderate correlations when |*r*| is between 0.4 and 0.7, and strong correlations when |*r*| is more than 0.7. Difference in alignment among the groups were assessed using a Kruskal-Wallis test. A *p*-value less than 0.05 was considered significant.

## Results

### Measurement results

The pre-operative HKA for the patient cohort was 174.10° ± 8.66° and the follow-up time was 16.94 ± 3.61 months. Joint alignment on the three measurement planes is shown in Table 1. On the coronal plane, the post-operative HKA was 179.46° ± 3.36° and presented as varus alignment, which was defined as an angle of less than 180°. The MDFA and MPTA were 89.08° ± 2.59° and 89.78° ± 1.68° respectively, which were similarly considered to be in varus alignment if the angle was less than 90°. On the sagittal plane, the FEA was 91.67° ± 2.18°, with an angle of more than 90° being considered extension. The TSA was 86.20° ± 1.77°, which represents a posterior slope when the angle is less than 90°. On the transverse plane, the FRA and TRA were 1.58° ± 2.83° and 3.32° ± 4.92° respectively, with positive values representing external alignment. The total HSS score and the score from each section are shown in Table 2.

**Table 1:** Measurement results of post-operative joint alignment.

	Mean	SD	Range
Pre-HKA (°)	174.1	8.7	156.1–198.6
Post-HKA (°)	179.5	3.4	173.2–187.5
MDFA (°)	89.1	2.6	83.8–93.5
MPTA (°)	89.8	1.7	86.0–93.6
FEA (°)	91.7	2.2	86.9–96.8
TSA (°)	86.2	1.8	82.9–89.8
FRA (°)	1.6	2.8	−5.6–7.5
TTA (°)	3.3	4.9	−7.7–12.6

**Table 2:** HSS scores of all patients.

HHS	Mean	SD	Range
Total	84.7	9.5	63.0–97.0
Pain	26.1	3.8	15.0–30.0
Function	18.3	3.3	8.0–22.0
ROM	12.2	2.2	5.0–16.0
Muscle strength	9.4	1.4	4.0–10.0
FE deformity	9.1	1.8	5.0–10.0
Stability	9.6	1.2	5.0–10.0

The outlier rates for all the post-operative alignments were calculated. The results showed that FEA had the highest rate (42%) in all alignments (Table 3).

**Table 3:** Outlier rates of component alignment (%).

HKA	MDFA	MPTA	FEA	TSA	FRA
40	34	10	42	18	36

### Linear correlation analysis of joint alignment and HSS scores

**Figure 4:**
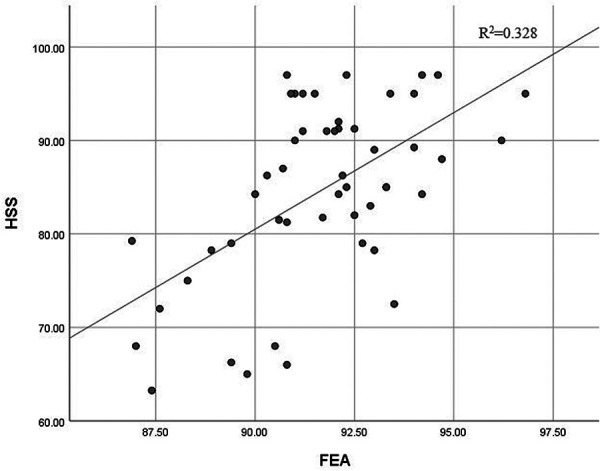
Correlation between HSS and FEA (HSS, hospital for special surgery knee scores; FEA, femoral flexion-extension angle).

The correlation coefficients (*r*) and associated *p*-values are shown in Table 4. The results show that the FEA had a positive linear correlation with the total HSS score (Figure 4, *r* = 0.572, *p* = 0.000), pain score (*r* = 0.347, *p* = 0.013), function score (*r* = 0.535, *p* = 0.000), ROM score (*r* = 0.368, *p* = 0.009), muscle score (*r* = 0.354, *p* = 0.012) and stability score (*r* = 0.312, *p* = 0.028). Larger values for MDFA were associated with lower FE deformity scores (*r* = −0.289, *p* = 0.042) and the TSA had a positive influence on the ROM scores (*r* = 0.436, *p* = 0.002). Also, it was found that different FRA would result in the different FE deformity scores (*r* = 0.312, *p* = 0.027), and the muscle strength score increased as the TRA increased (*r* = 0.402, *p* = 0.004). Positive correlations between results were confirmed when the *r*-value was greater than 0.

**Table 4:** Results of correlation analysis between alignments and clinical scores.

Independent	Dependent						
Total HSS	Pain	Function	ROM	Muscle strength	FE deformity	Stability
Post-HKA	*r*	−0.085	0.004	−0.062	0.001	−0.047	−0.101	−0.055
	*p*	0.558	0.980	0.669	0.995	0.744	0.484	0.702
MDFA	*r*	−0.228	−0.050	−0.016	−0.063	−0.153	**−0** **.** **289**	−0.135
	*p*	0.111	0.728	0.913	0.665	0.289	**0** **.** **042**	0.349
MPTA	*r*	−0.071	−0.063	−0.198	−0.007	−0.135	0.172	0.118
	*p*	0.626	0.666	0.168	0.963	0.352	0.232	0.412
FEA	*r*	**0** **.** **572**	**0** **.** **347**	**0** **.** **535**	**0** **.** **368**	**0** **.** **354**	0.258	**0** **.** **312**
	*p*	**<0** **.** **001**	**0** **.** **013**	**<0** **.** **001**	**0** **.** **009**	**0** **.** **012**	0.070	**0** **.** **028**
TSA	*r*	0.188	0.009	0.214	**0** **.** **436**	0.170	0.007	−0.106
	*p*	0.191	0.952	0.135	**0** **.** **002**	0.237	0.962	0.466
FRA	*r*	0.223	0.059	0.180	0.057	−0.008	**0** **.** **313**	0.183
	*p*	0.120	0.685	0.211	0.696	0.955	**0** **.** **027**	0.204
TRA	*r*	0.070	−0.007	−0.104	−0.143	**0** **.** **402**	0.201	0.063
	*p*	0.627	0.963	0.474	0.323	**0** **.** **004**	0.162	0.663

The bold values represents statistically significant results.

### Difference test results

The patients were divided into different groups according to the total HSS score. There were 28 patients in Group A, 16 patients in Group B, 6 patients in Group C. Patient age, pre-operative HKA, alignments on the three planes for the three groups were assessed using a Kruskal Wallis test. The results revealed significant differences in MDFA (*p* = 0.050) and FEA (*p* = 0.001) between the groups. Except for the parameters mentioned, no significant differences were detected between other measurements (*p* > 0.050). All results are detailed in Table 5.

**Table 5:** The results of the Kruskal-Wallis test between different groups.

	Group A		Group B		Group C		*p*
	Mean	SD	Mean	SD	Mean	SD	
Age	68.54	9.57	68.24	7.94	70.33	4.23	0.892
Pre-HKA	173.05	8.63	175.11	8.78	177.65	5.25	0.303
Post-HKA	178.97	2.67	180.00	4.13	181.57	4.39	0.413
MDFA	88.91	2.39	89.15	2.84	91.63	1.98	**0** **.** **050**
MPTA	89.54	1.20	90.16	2.17	89.93	2.52	0.499
FEA	92.62	1.66	90.50	2.24	89.15	1.59	**0** **.** **001**
TSA	86.31	2.07	86.18	1.21	85.70	1.63	0.858
FRA	2.08	2.14	0.81	3.49	−0.72	3.66	0.244
TRA	4.05	4.23	2.37	5.80	2.75	5.06	0.550

The bold values represents statistically significant results.

## Discussion

The main finding of this study was that there were significant differences in MDFA and FEA between the three groups with excellent, good and not good scores. It was also found that the total HSS only had a moderate correlation with FEA. Meanwhile, the outlier rate of FEA was the highest among all alignments within the same patient cohort.

This study found similar correlations between alignments and clinical outcomes as previous studies. Kastner et al. demonstrated that the sagittal alignment of femoral components is significantly correlated with the range of motion (36), which supports the associated between FEA and the ROM scores in this present study. Scott et al. found that the alignment of the femoral component on the sagittal plane was associated with anterior knee pain, and femoral component extension was a major risk factor (37). Similarly, this study found a significant correlation between FEA and pain score. Meanwhile, the functional scores had a moderate correlation with FEA, which is somewhat supported by Okamoto's study that good femoral sagittal alignment leads to better joint function (38). In this study, the FEA was also correlated with muscle strength and stability. Koh et al. reported that the quadriceps force, collateral ligament force and patella-femoral contact stress decreased as the angle between the axis of the femoral component and anterior cortex became smaller (39). This indicates that changes in the biomechanical environment around the knee may affect muscle strength and joint stability. Also, the significant correlation between FE-deformity scores and MDFA and FRA is supported by a study by Matziolis et al. detailing that distal femoral resection influences the flexion deformity of knee joint (40).

In this study, the FEA had the highest outlier rate, which is similar to findings from previous studies (Table 6). It is common for surgeons to attempt to align the femoral component when the cutting line is perpendicular to the anatomical axis on the sagittal plane and avoid the notch of the anterior cortical bone (48). However, it is not easy to identify the anatomical axis during conventional operations. When an intramedullary rod is used to represent the anatomical axis of the femur, errors with positioning of the entry point and insertion direction might lead to the rod being offset from the true axis. Such differences may also contribute to malalignment on the coronal plane. While variations in radiographic assessments could also influence the results, when using the anatomical axis and anterior cortical axis to evaluate femoral alignment on the sagittal plane, Jenny et al. reported little difference between the two axes and any variation in readings will likely have little clinical relevance (32). Tibial rotational alignment is considered one of the most controversial alignment methods in TKA because of the different reference landmarks used for the tibia and difference methods of alignment (4). This study performed a tibial trial during rotational alignment of the tibial component to confirm proper positioning, but this led to an unclear target of TRA value, and hence the outlier rate of TRA was not calculated.

**Table 6:** Comparison of outlier rates from published studies (%).

Year	Author	Cases	HKA	MDFA	MPTA	FEA	TSA	FRA
	Present study	50	40.00	34.00	10.00	42.00	18.00	36.00
2017	Ueyama (41)	75	-	13.30	4.00	21.30	8.00	-
2014	Huang (42)	37	27.10	27.10	2.70	43.20	0.00	10.90
2014	Chen (43)	72	27.80	50.00	25.00	36.10	16.70	-
2014	Kotela (44)	46	30.43	26.09	19.57	47.82	19.56	-
2014	Yan (45)	30	53.30	23.30	20.00	56.70	13.30	-
2014	Victor (46)	64	28.10	14.10	3.10	48.40	3.10	23.00
2013	Boonen (47)	82	18.00	13.00	2.00	65.00	28.00	-

The results showed a correlation between femoral component alignment on the sagittal plane (FEA) and complications after TKA, including pain, flexion contracture, and a restricted range of motion. A 10-year follow-up study by Scott et al. found sagittal plane positioning of the femoral component to be associated with long-term anterior knee pain, and it was suggested that femoral component extension might be a major risk factor for knee pain (37). Okamoto et al. found that FEA was significantly different between groups with differing degrees of flexion contracture, and they reported that FEA and body height were independent predictive risk factors for residual flexion contracture of more than 10° (38). Changes in the mechanical and kinematic environment in the knee joint resulting from different FEA were a likely reason for these complications.

The sagittal positioning of the femoral component is recognized as an important factor in knee joint mechanics and kinematics. It was reported that the femoral flexion-extension angle influenced the femorotibial contact position in the knee flexion which changed the arm of the quadriceps force and patellofemoral (PF) contact force (49). Using finite element models, Koh et al. investigated how variations in the FEA impacted knee mechanics and kinematics, and found the femorotibial contact points were positioned more posteriorly with larger FEA angles, and the quadriceps force, as well as the PF contact force, was reduced because of the decreased lever arm. It was suggested that placing the prosthesis in slight flexion could be an effective alternative technique to enable positive biomechanical effects with TKA (39, 50). Besides, the discrepancy of medial and lateral collateral ligaments between different femoral flexion-extension angles, the collateral ligament force decreased as the femoral component flexed during the knee bending. Large amounts of sagittal femoral component extension may be harmful to the collateral ligament. Errors in femoral component sagittal alignment contribute to imbalanced soft tissue that leads to instability and a limited range of motion (51). However, while these factors may improve clinical outcomes, they must also be considered when evaluating differences between studies and patient satisfaction.

Although digital techniques such as navigation, custom instrumentation and robotics have been commercially available and widely used for a number of years, conventional surgical techniques are still common because of the additional surgical time and costs associated with more advanced methods (27, 29, 30, 52). Moreover, mechanical alignment is regarded as one of the best approaches to TKA, with many studies demonstrating acceptable joint kinematics when the error of coronal alignment is less than 3°. However, contrasting studies have also reported that coronal alignment is not an accurate predictor of clinical outcomes and maintaining alignment within 0 ± 3° is not a “safe zone” when using more modern personalized alignment strategies (22, 53). The sagittal alignment for TKA, especially for the femoral component needs more consideration, and the surgical procedures and tools need to be improved to allow for more precise alignment.

There are also some limitations in this study. First, 50 TKA patients were enrolled in the study because of the considerable time and cost associated with patient examination. However, the sample size was sufficient according to the power analysis to assess the correlations between alignments and clinical scores. Moreover, the minimum follow-up time was 12 months. Future studies may consider longer-term follow-up.

## Conclusion

A significant and moderate correlation was found between FEA and the HSS score, and the FEA had the highest outlier rate. This suggests that FEA should be carefully considered when planning TKA and implant positioning. Surgical techniques and tools, especially for conventional surgery, need to be enhanced, to improve surgical accuracy and patient satisfaction.

## Data Availability

The original contributions presented in the study are included in the article/Supplementary Material, further inquiries can be directed to the corresponding author/s.
